# Differences in macular vessel density in the superficial plexus across cognitive impairment: the NORFACE cohort

**DOI:** 10.1038/s41598-022-21558-w

**Published:** 2022-10-08

**Authors:** Marta Marquié, Sergi Valero, Joan Martínez, Emilio Alarcón-Martín, Ainhoa García-Sánchez, Itziar de Rojas, Miguel Castilla-Martí, Luis Castilla-Martí, Isabel Hernández, Maitée Rosende-Roca, Liliana Vargas, Juan Pablo Tartari, Ester Esteban-De Antonio, Urszula Bojaryn, Vanesa Pytel, Leire Narvaiza, Montserrat Alegret, Gemma Ortega, Ana Espinosa, Ángela Sanabria, Alba Pérez-Cordón, Núria Lleonart, Nathalia Muñoz, Lluís Tárraga, Agustín Ruiz, Mercè Boada

**Affiliations:** 1 Ace Alzheimer Center Barcelona - Universitat Internacional de Catalunya, Barcelona, Spain; 2 Networking Research Center On Neurodegenerative Diseases (CIBERNED), Instituto de Salud Carlos III, Madrid, Spain; 3 Clínica Oftalmológica Dr. Castilla, Barcelona, Spain; 4 Department of Ophthalmology, Hospital del Mar and Hospital de l’Esperança – Parc de Salut Mar, Barcelona, Spain; 5 PhD Programme in Surgery and Morphological Sciences, Universitat Autònoma de Barcelona, Barcelona, Spain; 6 Hôpital Ophtalmique Jules-Gonin - Fondation Asiles des aveugles, University of Lausanne, Lausanne, Switzerland

**Keywords:** Alzheimer's disease, Diagnostic markers

## Abstract

Optical coherence tomography angiography (OCT-A) allows the detection of retinal vessel density (VD) loss, which is a reflection of brain vascular pathology. We aimed to investigate differences in macular VD in the superficial plexus in a large cohort of individuals cognitively unimpaired (CU), with mild cognitive impairment due to Alzheimer´s disease (MCI-AD), MCI due to cerebrovascular pathology (MCI-Va), probable Alzheimer´s disease dementia (ADD) and Vascular Dementia (VaD). Clinical, demographical, ophthalmological and OCT-A data from the *Neuro-ophthalmology Research at Fundació ACE* (NORFACE) project were analyzed. Differences of macular VD in four quadrants (superior, nasal, inferior and temporal) among the five diagnostic groups were assessed in a multivariate regression model, adjusted by age, sex, education, hypertension, diabetes mellitus, heart disease and stroke. The study cohort comprised 672 participants: 128 CU, 120 MCI-AD, 111 MCI-Va, 257 ADD and 56 VaD. Regression analysis showed a significantly higher VD in the temporal quadrant in MCI-AD compared to CU participants (49.05 ± 4.91 vs 47.27 ± 4.17, *p* = 0.02, d = 0.40), and a significantly lower VD in the inferior quadrant in MCI-Va compared to CU participants (48.70 ± 6.57 vs 51.27 ± 6.39, *p* = 0.02, d = 0.40). Individuals with heart disease presented significantly lower VD in the inferior quadrant than those without (*p* = 0.01). The interaction of sex and diagnosis had no effect in differentiating VD. Mini-Mental State Examination (MMSE) scores were not correlated to VD (all r < 0.16; *p* > 0.07). In conclusion, our study showed that the MCI-AD and MCI-Va groups had significant differences in macular VD in opposite directions in the temporal and inferior quadrants, respectively, compared to CU participants, suggesting that macular VD might be able to differentiate two pathogenic pathways (AD- and cerebrovascular-related) in early stages of cognitive decline.

## Introduction

Neuropathological studies suggest that “mixed dementia”, which involves the coexistence of Alzheimer´s disease (AD) pathology and brain vascular damage, is the most common underlying cause of cognitive impairment among the elderly^1^. In fact, cerebral small vessel disease and silent infarcts are present in up to 80% of dementia cases^2,3^. Epidemiological studies are possibly underestimating the contribution of brain vascular damage in dementias, due to the wrong classification of cases with vascular or mixed dementia as AD, especially in those studies without neuroimaging or neuropathology confirmation^4^.

Both neurodegeneration and brain vascular changes originate decades before the onset of cognitive symptoms and can be detected in vivo in the brains of cognitively normal individuals using different biomarkers such as brain magnetic resonance imaging (MRI)^5^, positron emission tomography (PET) with tracers against amyloid and tau^6^, and quantification of tau and amyloid levels in the cerebrospinal fluid (CSF)^7^. As these techniques are either quite expensive, invasive or not readily available, one of the main goals in the dementia research field is to develop novel inexpensive and non-invasive biomarkers. Currently, candidate markers are being evaluated in different fields such as genomics, plasma analytes and neuro-ophthalmology.

The retina is a projection of the central nervous system (CNS) through the optic nerve and thus is considered a “window to the brain”^8^. The retina is an attractive potential source of brain biomarkers, as it shares with the brain embryological, immunological and chemical features^8^. Unless the rest of the CNS, the retina can be directly visualized in vivo using Optical Coherence Tomography (OCT), an affordable, reliable, widely accessible and non-invasive imaging technique that obtains high-resolution tomographic micrometric images of the retina through the pupil^9^.

Structural OCT has been used for over three decades in the field of Ophthalmology to diagnose and monitor common ocular pathologies such as open-angle glaucoma, diabetic retinopathy or age-related macular degeneration^10^. OCT has also allowed the detection of thickness changes in different layers of the retina in neurological disorders such as optical neuritis, multiple sclerosis, Parkinson disease and AD^11^. OCT angiography (OCT-A) is a novel technique that allows the visualization of small retinal vessels and their blood flow without the need of intravenous contrast injection^12^. It obtains high resolution images of the retinal vasculature in 3D, in most cases approaching histological resolution^13^. Quantitative measures of retinal vascular features, such as vessel density (VD) assessed by OCT-A may detect areas of vascular loss that are not yet visible on fundus photographs^14^.

Several studies support the idea that retinal microvasculature damage reflects brain microvascular alterations^15^. The use of retinal vascular imaging such as OCT-A can provide critical information about the early pathophysiology of AD (and in particular, about its microvascular etiology) and the pathological mechanisms of brain vascular damage and its role as predictors of cognitive decline. So far, previous literature investigating OCT-A in cognitive disorders has shown discrepant results, with some small studies demonstrating differences in several vascular retinal measures across diagnostic groups^16,17^, while other did not^18^.

In this study we aimed to analyze the differences of vessel density (VD) in the macular superficial plexus as quantified by OCT-A in a large and well-characterized single-site cohort of individuals cognitively unimpaired (CU), with Mild Cognitive Impairment (MCI), probable Alzheimer´s disease dementia (ADD) and Vascular dementia (VaD) who were evaluated in a Memory Clinic, along with the effects of sex and the correlation of macular VD with cognitive measures.

## Results

### Demographic and clinical characteristics of the cohort

**Figure 1:**
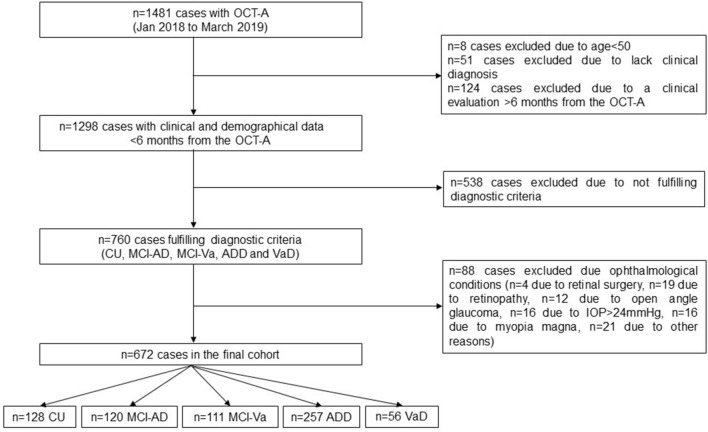
Participants’ selection algorithm. CU = cognitively unimpaired; ADD = probable Alzheimer´s disease dementia; IOP = intraocular pressure: MCI-AD = mild cognitive impairment due to Alzheimer´s disease; MCI-Va = mild cognitive impairment due to cerebrovascular pathology; OCT-A = optical coherence tomography angiography; VaD = vascular dementia.

Data from 1481 individuals with available clinical information and OCT-A obtained between January 2018 and March 2019 were initially reviewed. Several exclusion criteria were applied: age < 50yo (n = 8), lack of finalized clinical diagnosis (n = 51), lack of a clinical evaluation within 6 months from the OCT-A (n = 124), not fulfilling the diagnostic group criteria (CU, MCI-AD, MCI-Va, ADD, VaD) (n = 538), and finally, ophthalmological conditions that could interfere with the OCT-A measurements (n = 88; n = 4 due to retinal surgery, n = 19 due to retinopathy, n = 12 due to open angle glaucoma, n = 16 due to IOP > 24 mmHg, n = 16 due to myopia magna, n = 21 due to other reasons) (see the participants’ algorithm selection in Fig. 1).

The final sample consisted of 672 individuals with the following diagnosis: 128 CU, 120 MCI-AD, 111 MCI-Va, 257 ADD and 56 VaD. Demographic characteristics and past medical history of the cohort are displayed in Table 1.

**Table 1:** Demographic and clinical characteristics of the cohort.

	Diagnostic group	Mean	SD	Intergroup significance
Age (years)	CU (n = 128)	65.44	7.23	< 0.001^a^
	MCI-AD (n = 120)	75.98	7.05	
	MCI-Va (n = 111)	77.08	7.34	
	ADD (n = 257)	80.39	7.29	
	VaD (n = 56)	80.52	8.11	
	Total (n = 672)	76.05	9.46	
Sex (% women)	CU (n = 128)	69.5%	n/a	< 0.001^b^
	MCI-AD (n = 120)	46.7%	n/a	
	MCI-Va (n = 111)	52.3%	n/a	
	ADD (n = 257)	70.4%	n/a	
	VaD (n = 56)	62.5%	n/a	
	Total (n = 672)	64.3%	n/a	
Education (years)	CU (n = 128)	11.91	4.37	< 0.001^a^
	MCI-AD (n = 120)	8.00	4.50	
	MCI-Va (n = 111)	7.41	3.80	
	ADD (n = 257)	6.35	3.99	
	VaD (n = 56)	6.27	4.00	
	Total (n = 672)	7.96	4.75	
MMSE (score)	CU (n = 128)	29.13	0.92	< 0.001^a^
	MCI-AD (n = 120)	24.73	3.09	
	MCI-Va (n = 111)	26.13	2.64	
	ADD (n = 257)	19.06	4.61	
	VaD (n = 56)	21.91	5.95	
	Total (n = 672)	22.86	5.65	
Hypertension	CU (n = 128)	35.2%	n/a	< 0.001^b^
	MCI-AD (n = 120)	53.3%	n/a	
	MCI-Va (n = 111)	67.6%	n/a	
	ADD (n = 257)	63.0%	n/a	
	VaD (n = 56)	83.9%	n/a	
	Total (n = 672)	56.7%	n/a	
Diabetes mellitus	CU (n = 128)	7.8%	n/a	< 0.001^b^
	MCI-AD (n = 120)	12.5%	n/a	
	MCI-Va (n = 111)	26.1%	n/a	
	ADD (n = 257)	13.6%	n/a	
	VaD (n = 56)	41.1%	n/a	
	Total (n = 672)	14.8%	n/a	
Dyslipidemia	CU (n = 128)	40.6%	n/a	0.19^b^
	MCI-AD (n = 120)	53.3%	n/a	
	MCI-Va (n = 111)	50.5%	n/a	
	ADD (n = 257)	49.4%	n/a	
	VaD (n = 56)	57.1%	n/a	
	Total (n = 672)	49.0%	n/a	
Heart disease	CU (n = 128)	10.9%	n/a	< 0.001^b^
	MCI-AD (n = 120)	26.7%	n/a	
	MCI-Va (n = 111)	29.7%	n/a	
	ADD (n = 257)	25.7%	n/a	
	VaD (n = 56)	44.6%	n/a	
	Total (n = 672)	24.4%	n/a	
COPD	CU (n = 128)	7.8%	n/a	0.18^b^
	MCI-AD (n = 120)	9.2%	n/a	
	MCI-Va (n = 112)	15.3%	n/a	
	ADD (n = 257)	8.2%	n/a	
	VaD (n = 56)	14.3%	n/a	
	Total (n = 672)	8.9%	n/a	
Stroke	CU (n = 128)	3.1%	n/a	< 0.001^b^
	MCI-AD (n = 120)	5%	n/a	
	MCI-Va (n = 111)	19.8%	n/a	
	ADD (n = 257)	5.8%	n/a	
	VaD (n = 56)	25%	n/a	
	Total (n = 672)	7%	n/a	
Smoking	CU (n = 128)	7.8%	n/a	0.23^b^
	MCI-AD (n = 120)	2.5%	n/a	
	MCI-Va (n = 111)	9%	n/a	
	ADD (n = 257)	4.7%	n/a	
	VaD (n = 56)	7.1%	n/a	
	Total (n = 672)	5.2%	n/a	

Demographic and medical conditions among groups are summarized.

^a^1-factor ANOVA

^b^Pearson’s Chi2 test.

ADD = probable Alzheimer´s disease dementia; COPD = chronic obstructive pulmonary disease; CU = cognitively unimpaired; MCI-AD = mild cognitive impairment due to Alzheimer´s disease; MCI-Va = mild cognitive impairment due to cerebrovascular pathology; MMSE = Mini Mental State Examination; n/a = not available; SD = standard deviation; VaD = vascular dementia.

Significance was set up at *p* < 0.05.

### Multinomial regression analysis of demographic, clinical and structural OCT variables among diagnostic groups

The first multinomial regression analysis exploring the distribution of age, sex and education among the diagnostic groups showed that these three demographical variables had a significant effect, at least in one contrast, so they were all included as adjusting factors in the final analysis (Supplementary Table S1).

The second multinomial regression analysis exploring several cardiovascular conditions among diagnostic groups showed that hypertension, diabetes mellitus, heart disease and stroke were the only factors with at least a significant discrepancy in one contrast among the diagnostic groups, so those were also included as adjusting factors in the final analysis. (Supplementary Table S2).

### Multivariate regression analysis of macular VD differences among diagnostic groups

**Figure 2:**
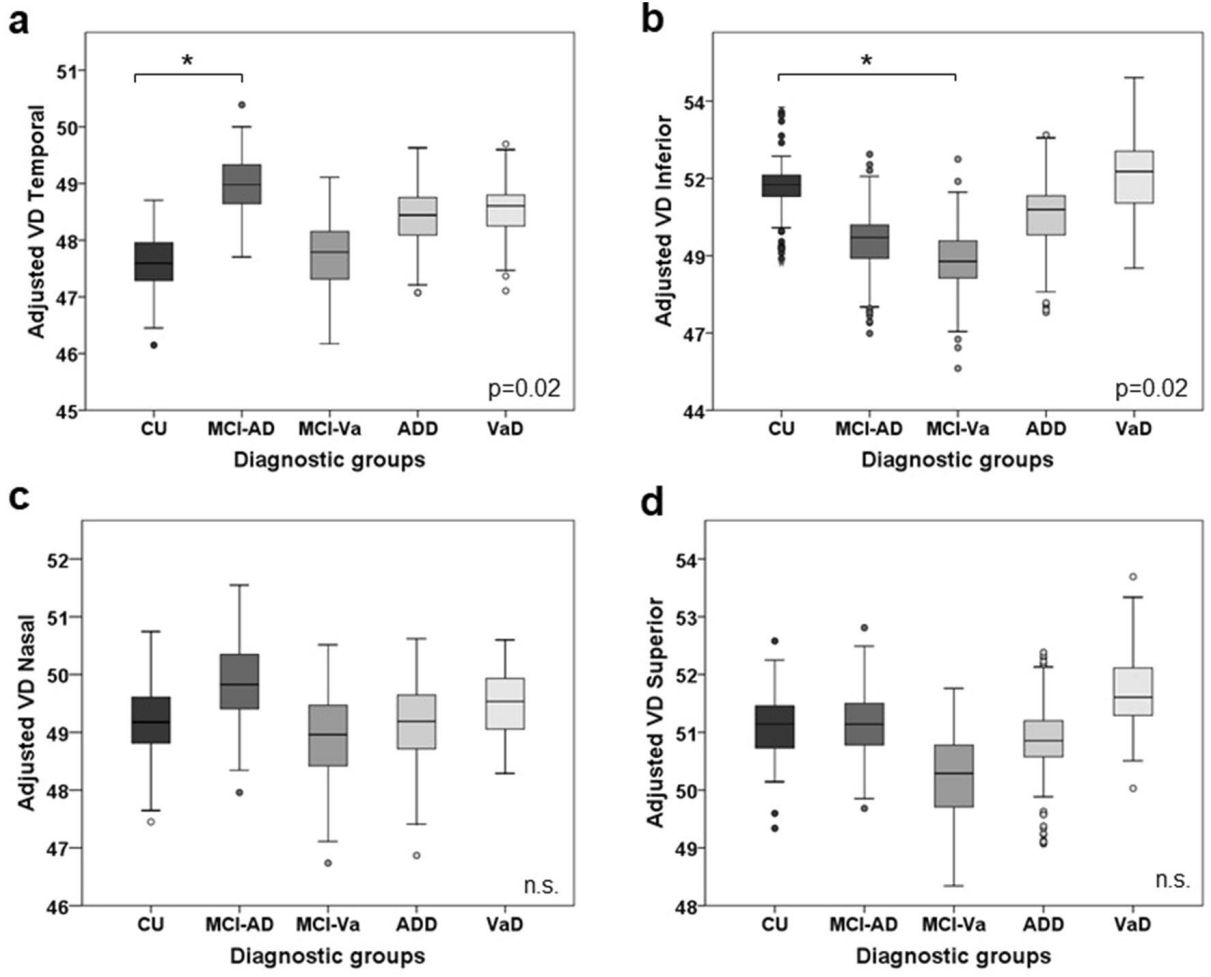
Adjusted macular VD measurements by diagnostic group. Macular VD differences among diagnostic groups in (**a**) temporal, (**b**) inferior, (**c**) nasal and (**d**) superior quadrants. Macular VD measurements are adjusted by age, sex, education, hypertension, diabetes mellitus, heart disease and stroke. CU = cognitively unimpaired; ADD = probable Alzheimer´s disease dementia; MCI-AD = mild cognitive impairment due to Alzheimer´s disease; MCI-Va = mild cognitive impairment due to cerebrovascular pathology; n.s. = non-significant.; VaD = vascular dementia; VD = vessel density. Statistical significance was set-up at *p* < 0.05.

Table 2 depicts the contribution of each adjusting factor and diagnosis to macular VD variance. Regression models revealed a significant effect of diagnosis on VD values. On one hand, MCI-AD participants showed a significantly higher macular VD in the temporal quadrant compared to CU individuals (49.05 ± 4.91 vs 47.27 ± 4.17, *p* = 0.02, d = 0.40) (Fig. 2a). Additionally, MCI-Va participants showed a significantly lower macular VD in the inferior quadrant compared to CU individuals (48.70 ± 6.57 vs 51.27 ± 6.39, *p* = 0.02, d = 0.40) (Fig. 2b). On the other hand, no significant differences in macular VD nasal (Fig. 2c) and VD superior (Fig. 2d) quadrant measurements were detected among diagnostic groups. Lastly, the presence of heart disease showed a significant inverse relationship with VD also in the inferior quadrant (*p* = 0.01), so that participants with heart disease had significantly lower VD in the inferior quadrant compared to those without this condition. In contrast, age, sex, education, hypertension, diabetes mellitus and stroke presented no significant contribution to VD variability.

**Table 2:** Multivariate regression analysis of macular VD measurements.

Covariates	Dependent variables	Coefficient	t	Significance	Beta
Sex	VD nasal	0.03	0.06	0.96	0.01
	VD superior	0.10	0.16	0.88	0.01
	VD temporal	0.56	1.25	0.21	0.05
	VD inferior	0.70	1.12	0.27	0.05
Age	VD nasal	− 0.05	− 1.37	0.17	− 0.07
	VD superior	− 0.06	− 1.46	0.15	− 0.07
	VD temporal	− 0.05	− 1.82	0.07	− 0.09
	VD inferior	− 0.02	− 0.40	0.70	− 0.02
Education	VD nasal	− 0.11	− 1.78	0.08	− 0.08
	VD superior	0.01	0.14	0.89	0.01
	VD temporal	− 0.05	− 1.00	0.32	− 0.05
	VD inferior	− 0.06	− 0.89	0.38	− 0.04
Hypertension	VD nasal	− 0.25	− 0.47	0.64	− 0.02
	VD superior	0.12	0.20	0.84	0.01
	VD temporal	0.15	0.34	0.73	0.01
	VD inferior	0.59	0.93	0.35	0.04
Diabetes mellitus	VD nasal	− 0.38	− 0.56	0.58	− 0.02
	VD superior	0.88	1.15	0.25	0.05
	VD temporal	0.33	0.57	0.57	0.02
	VD inferior	1.57	1.95	0.05	0.08
Heart disease	VD nasal	− 0.64	− 1.09	0.27	− 0.04
	VD superior	0.33	0.51	0.61	0.02
	VD temporal	0.44	0.88	0.38	0.04
	VD inferior	− 2.11	− 3.06	0.01*	− 0.13
Stroke	VD nasal	0.19	0.21	0.83	0.01
	VD superior	− 1.32	− 1.36	0.18	− 0.06
	VD temporal	− 0.46	− 0.63	0.53	− 0.03
	VD inferior	0.99	0.96	0.34	0.04
Diagnostic groups: CU vs MCI-AD	VD nasal	0.87	0.98	0.33	0.05
	VD superior	0.64	0.65	0.52	0.04
	VD temporal	1.77	2.36	0.02*	0.13
	VD inferior	− 1.59	− 1.52	0.13	− 0.08
Diagnostic groups: CU vs MCI-Va	VD nasal	0.07	0.07	0.94	0.01
	VD superior	− 0.22	− 0.21	0.84	− 0.01
	VD temporal	0.50	0.64	0.52	0.04
	VD inferior	− 2.58	− 2.34	0.02*	− 0.13
Diagnostic groups: CU vs ADD	VD nasal	0.27	0.31	0.76	0.02
	VD superior	0.57	0.59	0.55	0.04
	VD temporal	1.23	1.68	0.09	0.11
	VD inferior	− 1.04	− 1.03	0.31	− 0.07
Diagnostic groups: CU vs VaD	VD nasal	0.85	0.73	0.47	0.04
	VD superior	1.40	1.07	0.29	0.06
	VD temporal	1.29	1.30	0.19	0.07
	VD inferior	0.09	0.07	0.95	0.01

The multivariate regression analysis included the following adjusting factors: age, sex, years of education, hypertension, diabetes mellitus, heart disease and stroke.

ADD = probable Alzheimer´s disease dementia; CU = cognitively unimpaired; MCI-AD = mild cognitive impairment due to Alzheimer´s disease; MCI-Va = mild cognitive impairment due to cerebrovascular pathology; VaD = vascular dementia; VD = vessel density.

Significance was set up at *p* < 0.05.

**Figure 3:**
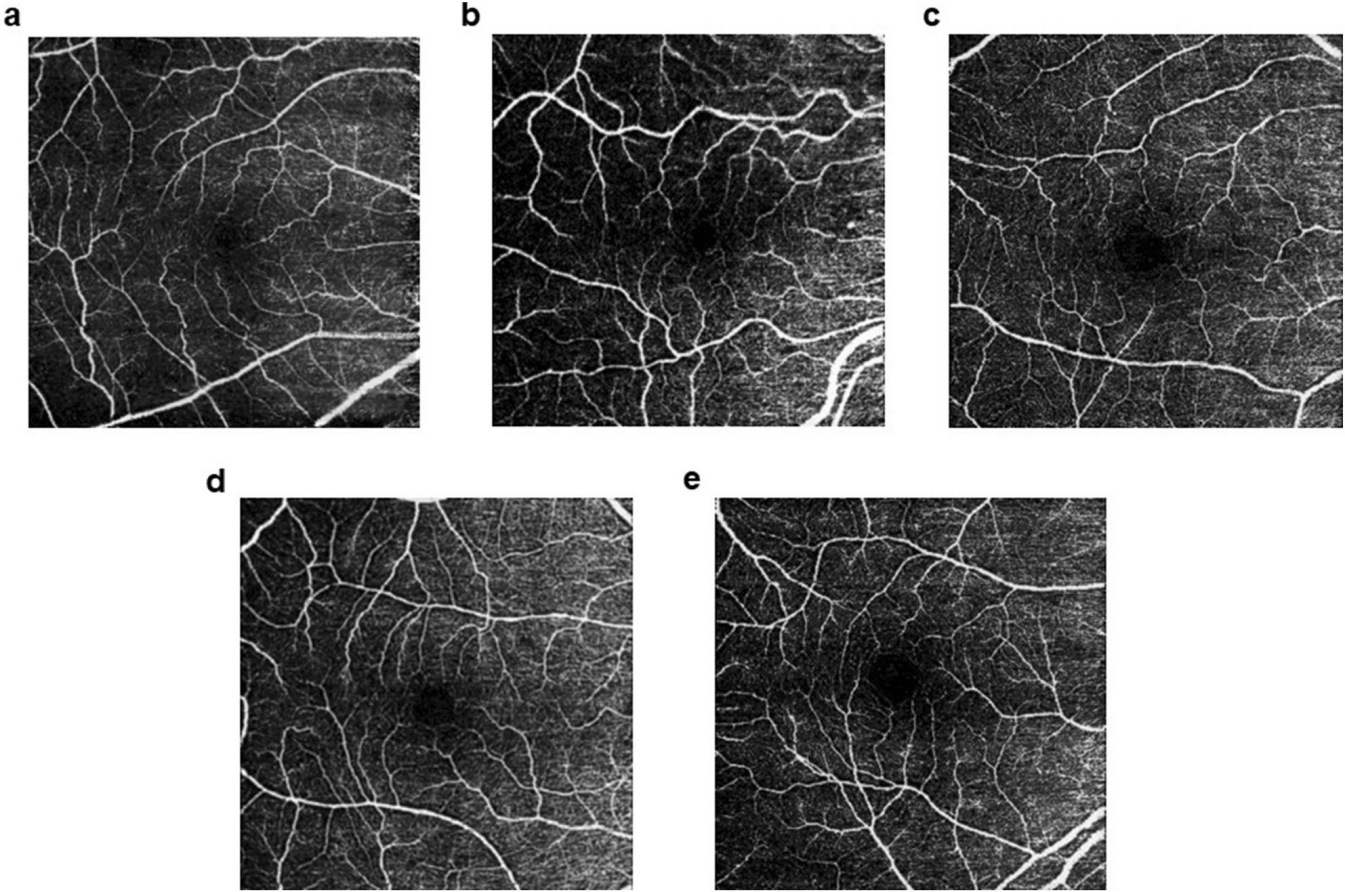
Representative VD images from the superficial vascular plexus at the macular region for each diagnostic group: CU (**a**), MCI-AD (**b**), MCI-Va (**c**), ADD (**d**) and VaD (**e**). CU = cognitively unimpaired; ADD = probable Alzheimer´s disease dementia; MCI-AD = mild cognitive impairment due to Alzheimer´s disease; MCI-Va = mild cognitive impairment due to cerebrovascular pathology; VaD = vascular dementia; VD = vessel density.

Representative VD images from the superficial vascular plexus at the macular region for each diagnostic group are depicted in Fig. 3.

Raw and adjusted sector-specific macular VD measures across diagnostic groups are displayed in Table 3.

**Table 3:** Raw and adjusted macular VD differences across diagnostic groups.

Group (n)	Mean	SD	Mean^aa^	SEM^aa^
**VD nasal**				
CU (n = 128)	49.18	5.52	48.94	0.68
MCI-AD (n = 120)	49.81	6.14	49.81	0.57
MCI-Va (n = 111)	48.95	7.16	49.01	0.60
ADD (n = 257)	49.17	5.87	49.21	0.41
VaD (n = 56)	49.51	6.67	49.78	0.87
**VD superior**				
CU (n = 128)	51.10	5.04	50.52	0.76
MCI-AD (n = 120)	51.18	6.09	51.16	0.64
MCI-Va (n = 111)	50.21	6.93	50.30	0.67
ADD (n = 257)	50.88	7.63	51.09	0.46
VaD (n = 56)	51.76	8.32	51.92	0.97
**VD temporal**				
CU (n = 128)	47.58	4.17	47.27	0.58
MCI-AD (n = 120)	48.97	4.91	49.05	0.48
MCI-Va (n = 111)	47.71	4.98	47.78	0.51
ADD (n = 257)	48.42	5.53	48.50	0.35
VaD (n = 56)	48.55	6.80	48.56	0.73
**VD inferior**				
CU (n = 128)	51.20	6.39	51.27	0.80
MCI-AD (n = 120)	49.41	6.95	49.68	0.67
MCI-Va (n = 111)	48.86	6.57	48.70	0.71
ADD (n = 257)	50.25	8.10	50.23	0.49
VaD (n = 56)	51.69	7.60	51.37	1.02

Raw and adjusted macular VD means, standard deviation (SD) and standard error of the mean (SEM) are shown. Dispersion is shown as SEM.

^aa^ = after adjustment for the following factors: age, sex, years of education, hypertension, diabetes mellitus, heart disease and stroke, using multivariate regression analysis.

ADD = probable Alzheimer´s disease dementia; CU = cognitively unimpaired; MCI-AD = mild cognitive impairment due to Alzheimer´s disease; MCI-Va = mild cognitive impairment due to cerebrovascular pathology; SD = standard deviation; SEM = standard error of the mean; VaD = vascular dementia; VD = vessel density.

Statistical significance was set up at *p* < 0.05.

The above regression models showed similar results without including cardiovascular conditions as adjusting factors (Supplementary Table S3).

Additionally, we rerun the multivariate regression analysis for every macular VD quadrant without including the outliers cases (defined as ± 3 standard deviations of the VD mean). The number of outlier cases detected per macular VD quadrant was the following: VD Nasal: n = 12 (8 upper limit, 4 lower limit), VD Superior: n = 14 (5 upper limit, 9 lower limit), VD Temporal: n = 11 (9 upper limit, 2 lower limit) and VD Inferior: n = 13 (8 upper limit, 5 lower limit). Without including outliers cases, the multivariate regression analysis showed similar significant results of the comparison of CU vs MCI-AD groups in the VD temporal quadrant (*p* = 0.02), CU vs MCI-Va groups in the VD inferior quadrant (*p* = 0.03), and presence vs absence of heart disease in the VD inferior quadrant (*p* < 0.01). Additionally, now the ADD group showed higher VD in the temporal quadrant compared to the CU individuals (*p* = 0.05), and age showed an inverse association with VD in the nasal and inferior quadrants (*p* = 0.05 and *p* = 0.04, respectively).

Lastly, pairwise comparisons were executed using Tukey correction under an exploratory aim for each VD measure separately, comparing every diagnostic group with the rest of categories. None of the contrasts presented a significant effect (Supplementary Table S4).

### Sex effect in macular VD across diagnostic groups

The interaction of sex and diagnosis had no effect in differentiating VD measurements, adjusted by age, education, hypertension, diabetes mellitus, heart disease and stroke (Supplementary Table S5). Thus, differences in macular VD among diagnostic groups were not significantly influenced by sex.

### Macular VD association with Mini-Mental Scale Examination (MMSE) scores

MMSE scores did not show significant correlations with any of the four macular VD measurements analyzed, either in the whole sample or in any of the five diagnostic groups separately, adjusted by age, sex, years of education, hypertension, diabetes mellitus, heart disease and stroke (all r < 0.16; *p* > 0.07) (see Table 4).

**Table 4:** Partial correlation of macular VD measurements with MMSE scores.

Diagnostic groups	Variable	r	Significance
Whole sample (n = 672)	VD nasal	0.01	0.97
	VD superior	0.04	0.27
	VD temporal	− 0.03	0.39
	VD inferior	− 0.01	0.83
CU (n = 128)	VD nasal	0.03	0.71
	VD superior	0.05	0.62
	VD temporal	0.04	0.67
	VD inferior	− 0.07	0.43
MCI-AD (n = 120)	VD nasal	− 0.17	0.07
	VD superior	0.10	0.31
	VD temporal	− 0.09	0.92
	VD inferior	0.16	0.09
MCI-Va (n = 111)	VD nasal	0.10	0.33
	VD superior	0.14	0.15
	VD temporal	− 0.12	0.23
	VD inferior	− 0.13	0.20
ADD (n = 257)	VD nasal	− 0.06	0.35
	VD superior	0.07	0.27
	VD temporal	0.03	0.66
	VD inferior	− 0.04	0.52
VaD (n = 56)	VD nasal	− 0.06	0.69
	VD superior	− 0.01	0.99
	VD temporal	0.06	0.68
	VD inferior	0.11	0.46

The model included the following adjusting factors: age, sex, years of education, hypertension, diabetes mellitus and stroke.

ADD = probable Alzheimer´s disease dementia; CU = cognitively unimpaired; MCI-AD = mild cognitive impairment due to Alzheimer´s disease; MCI-Va = mild cognitive impairment due to cerebrovascular pathology; VaD = vascular dementia; VD = vessel density.

Significance was set up at *p* < 0.05.

## Discussion

In this study we investigated differences of macular VD in the superficial plexus assessed by OCT-A within a large cohort of individuals with different degrees of cognitive impairment (CU, MCI-AD, MCI-Va, ADD and VaD) who were evaluated in a Memory Unit. Our results highlighted that the two MCI groups (MCI-AD and MCI-Va) showed significant differences in opposite directions in macular VD in the temporal and inferior quadrants, respectively, compared to CU participants, suggesting that VD might be able to differentiate two pathogenic pathways (AD- and cerebrovascular-related) in early stages of cognitive decline. No significant effect of the interaction of sex and diagnosis in predicting macular VD was detected and lastly, macular VD did not correlate with MMSE scores.

In the dementia field it is essential to investigate novel biomarkers linked to early AD pathology and concomitant microvascular changes, in order to develop better diagnostic and therapeutic options. The early identification of cerebrovascular pathology is very relevant, as asymptomatic brain vascular changes in middle-aged adults are associated with a higher risk of future cognitive decline and disability^19^. The NORFACE study was set up at Ace Alzheimer Center Barcelona back in 2014 in order to search for retinal biomarkers related to cognitive decline by using OCT. Our previous works analyzing peripapillary and macular thickness and volumes in a large cohort of patients from our Memory Unit did not detect significant differences among CU, MCI and ADD individuals^20,21^. Further, in a group of 129 participants with subjective cognitive decline (SCD) from the FACEHBI cohort, we found that thickening of the inner nasal macular region was associated with higher brain amyloid deposition measured by 18F-Florbetaben-PET but not with progression to MCI after a 2-year follow-up^22^. After these initial studies focused on retinal structural measurements, we moved to explore the vascular component as a potential biomarker of cognitive decline. In order to do that, we used the OCT-A technique, which is a non-expensive and non-invasive method of assessing retinal vascular characteristics. OCT-A can pinpoint the 3D vasculature of different retinal layers, calculate the area of the foveal avascular zone (FAZ) and measure the density of the capillary plexuses, thus allowing quantitative evaluation of retinal microvascular perfusion and retinal morphology^17^.

In the present study we were able to detect early changes of macular VD in the MCI-AD and MCI-Va groups compared to CU individuals, but in opposite directions and different locations. First, the MCI-AD participants showed significantly higher macular VD in the temporal quadrant compared to CU individuals, after controlling for age, sex, education, hypertension, diabetes mellitus, heart disease and stroke. The two dementia groups (ADD and VaD) also exhibited higher VD in the temporal quadrant than CU individuals, although interestingly, these differences did not reach statistical significance. Several concomitant phenomena occurring during the progression of cognitive impairment (from MCI to dementia) could be playing a part in this, such as the onset of new medications or the appearance of other systemic and brain pathologies. Additionally, VD measures in the nasal quadrant also showed a similar trend of higher VD in the MCI-AD group compared to CU participants. Similar to our data, two publications demonstrated increase in different retinal vascular parameters in individuals with early stages of AD-related cognitive impairment compared to controls, but with smaller samples. First, van de Kreeke et al. reported a significantly higher VD in both peripapillary and macular regions in 13 Aβ + compared to 111 Aβ- cognitively healthy twins, but no differences in the FAZ size^23^. Biscetti et al. showed higher measures of fractal dimension in a group of 24 MCI with a CSF AD-profile compared to a group of 13 controls^24^.

Although it is well known that brain vascular damage and neurodegeneration develop in parallel, the earliest brain microvascular changes that predict future development of dementia are still elusive, as well as the specific patterns of retinal vascular changes that develop in parallel in different vascular and neurodegenerative conditions. One potential explanation for the finding of a higher VD in MCI-AD compared to CU individuals detected in our study is the recruitment of new retinal vessels and increased blood flow related to the neuroinflammation phenomena, that occurs as a compensatory mechanism in response to early vascular dysregulation (amyloid angiopathy, hypoxia, non-productive angiogenesis) taking place in the initial stages of AD (MCI)^25–28^. These newly recruited retinal vessels become visible and cause a stronger vascular signal (higher VD) in OCT-A in MCI-AD participants, compared to that detected in CU individuals. Then, as neuroinflammation and amyloid accumulation further develop through the dementia stage, damage of retinal vasculature is established, with a subsequent reduction of VD detectable on OCT-A. In our cohort, the two dementia groups (ADD and VaD) showed non-significant lower VD compared to MCI-AD participants in the temporal quadrant, but these VD values were still higher than that in CU individuals. It is important to note that although in our study the MCI-AD group lacked biomarker confirmation for AD, we selected only MCI individuals with a “probable amnestic” profile, which is the clinical phenotype more strongly associated to AD^29^. New studies comparing VD to AD-related biomarker data such as CSF and PET are necessary to disentangle this point.

Data from other studies analyzing the relationship between retinal vascular pathology and cognitive decline, mostly using much smaller samples than ours and focusing on data from the deep retinal vascular plexus, largely point to retinal vascular loss in MCI and dementia patients compared to healthy controls, contrary to our current findings in the MCI-AD group. Wu et al. analyzed several retinal microvascular parameters in 21 healthy controls, 21 MCI patients and 18 ADD patients, showing that both the ADD and MCI groups had retinal microvascular loss compared to the control group, in particular reduced VD especially in the deep capillary plexus and enlargement of the FAZ^16^. This study also demonstrated that early changes in the inner annular zone of the deep retinal capillary plexus were already present in the MCI stage, while more widespread changes appeared later in ADD^16^. Zabel et al. compared OCT-A findings in a cohort of 27 mild and moderate ADD patients, 27 open-angle glaucoma and 27 cognitively healthy controls, showing different retinal regional patterns of microcirculatory impairment in ADD and open-angle glaucoma^30^. The study highlighted that ADD patients had reduced VD in the deep vascular plexus and enlarged FAZ compared to glaucoma patients and controls, while glaucoma patients had reduced VD in radial peripapillary capillaries and superficial vascular plexus compared to ADD patients and controls^30^. Jiang et al. showed similar results in a cohort of 12 ADD patients, 19 MCI patients and 21 controls^17^. Biscetti et al. showed a significant reduction in both vascular perfusion density and vessel length density in a group of 24 MCI with a CSF AD-profile compared to a group of 13 controls^24^. Querques et al. used dynamic vessel analyzer in a cohort of ADD, MCI and controls, and showed that the retinal neurovascular coupling was significantly impaired in ADD and MCI compared to controls^18^. In particular, the arterial dilation in response to flicker light was decreased in the ADD group compared to controls, while the reaction amplitude was decreased in both ADD and MCI groups compared to controls. Moreover, CSF Aβ levels were directly correlated with both the arterial dilation and reaction amplitude. Finally, no OCT-A variables (perfusion density) differed among groups. Zhang et al. pointed that patients with amnestic MCI/early ADD presented decreased parafoveal VD and flow compared to cognitively normal controls^31^. Grewal et al. described OCT-A features of a pair of 96-year old female monozygotic twins, showing that the twin with advanced ADD had a significantly reduced VD and larger FAZ in the superficial capillary plexus as well as a thinner choroid compared to the cognitively normal twin^32^. Two other studies showed that a lower retinal VD was associated with AD, suggesting that these retinal changes were reflecting similar ones occurring in the brain microvasculature^33,34^.

Our data also highlighted specific regional VD changes in the inferior quadrant associated to cerebrovascular pathology and risk factors. First, the MCI-Va group showed significantly lower macular VD in the inferior quadrant compared to CU individuals. In fact, the MCI-Va group showed the lowest VD measures in all macular quadrants, but these differences only reached significance in the inferior quadrant. Interestingly, the VaD group did not show significant VD changes in this region compared to CU participants. Additionally, individuals with heart disease displayed a significant lower VD only in the inferior quadrant compared to those without this condition. Few studies have incorporated patients with cerebrovascular cognitive impairment when studying retinal vascular changes using OCT-A. The AGES- Reykjavik Study concluded that retinopathy was associated with VaD but not with ADD, and that the risk of developing dementia was increased in those individuals with retinopathy and brain microinfarcts, suggesting that a high burden of vascular damage in both retina and brain was related to more severe cognitive consequences^35^. The relationship between retinal vascular damage and VaD could not be established in the Cardiovascular Health Study neither in the Rotterdam Study, due to the low number of cases evaluated^36,37^.

Regarding the relationship between retinal vascular parameters and cognitive performance, in our large cohort, none of the four macular VD measurements were significantly associated with MMSE scores across the whole sample or in any of the diagnostic groups separately. Several other studies have also analyzed this subject, using much smaller samples, most of them showing significant associations between retinal vascular changes and cognition, contrary to our results. Zhang et al. showed that in a cohort of 32 individuals with MCI, early ADD and normal cognition, lower MoCA scores were correlated with lower capillary density in the macular and optic nerve regions across groups^31^. Similarly, Bulut et al. reported positive correlations between vascular density and MMSE in a cohort of 52 ADD and cognitively normal participants^38^. Jiang et al. reported a positive correlation between MMSE scores and VD in a group of 19 MCI patients, but not in a group of 12 ADD patients^17^. Ashimatey et al. analyzed the association of capillary perfusion (vessel skeleton density) with cognitive scores and MRI-derived cerebrovascular perfusion in a cohort of 61 elderly latinx subjects at high risk for vascular cognitive impairment^39^. In this study, lower capillary perfusion was associated with higher CDR-SOB and lower visuospatial/executive MoCA scores, along with lower cerebrovascular reactivity and middle cerebral artery perforator perfusion^39^. On the contrary, Zabel et al. did no detect any association between MMSE scores and retinal VD in a cohort of 27 patients with ADD^30^.

Regarding the comparison of data from different studies, it is worth mentioning that OCT-A measurements of VD, perfusion density and FAZ dimensions are not interchangeable across different OCT-A platforms^40^. Moreover, it is suspected that the reproducibility of measurements could be lower in eyes with retinal microvasculature abnormalities and are also impacted by the OCT-A scan size^41,42^.

We acknowledge that our study has several limitations. First of all, the VaD group had a relatively small size compared to the rest of diagnostic groups. Second, our results were cross-sectional, not being able to show changes over time in macular VD measures or the predictive value of baseline macular VD in cognitive changes. Third, our VD measurements were limited to the macular region and the superficial vascular plexus, and we lacked information about FAZ changes and VD in the deep plexus, which other studies have found to be associated to cognitive decline^16,18,32,43,44^. Forth, no MRI measures of brain vascular pathology were available to compare with those of retinal vascular pathology. Fifth, we lacked information about cardiovascular-related drugs taken by the study participants, which could potentially affect VD. Also, we lacked information about the quality of the OCT-A images and its potential differences across the diagnostic groups. Lastly, we did not have AD-related biomarker data to confirm the clinical diagnosis and correlate with VD measures.

We also consider that our study has several strengths compared to previous works. First, our cohort consisted of a large and single-site sample of consecutive individuals who consulted to a Memory Unit due to cognitive concerns. Importantly, we performed the same comprehensive cognitive evaluation and standardized protocol to all participants, including CU individuals. This differentiates our study from others, which used much simpler tests for the diagnosis or were not well detailed, especially for those participants labeled as healthy controls^18,30^. We limited our analysis to data from the right eye, while others groups used both eyes from the same individual as separate data^16,18,44^. Our study included MCI-Va and VaD groups, while most of OCT-A publications lacked those. Our participants’ age range was quite large (50–99), allowing us to potentially detect macular VD changes in early and late ages. Notably, we used age, sex, education, hypertension, diabetes mellitus, heart disease and stroke as covariates in all our analyses and investigated the effect of sex in our results. Lastly, the neurologist and optometrist were blinded of each other’s diagnosis.

In summary, our study detected significant changes of macular VD at the superficial plexus in opposite directions in the MCI-AD and MCI-Va groups compared to CU participants in the temporal and inferior quadrants, respectively. These results indicate that VD assessed by OCT-A might be a biomarker able to differentiate two pathogenic pathways (AD- and cerebrovascular-related) in early stages of cognitive decline (MCI), while these differences disappear during the progression of the disease (ADD and VaD) as pathologies overlap and evolve in different ways. Additionally, our study detected a lack of correlation between macular VD measurements and MMSE scores and no effect of the interaction of sex and diagnostic group in differentiating VD measurements. Further studies are needed to investigate the usefulness of OCT-A to assess retinal vascular changes in large cohorts of individuals with different degrees of cognitive decline in the AD spectrum and VaD. It is also relevant to compare OCT-A measures with brain MRI changes and AD-related biomarkers (CSF and PET), which we plan to investigate in the near future using additional data from the NORFACE project and related studies at Ace Alzheimer Center Barcelona. We believe that the validation of OCT-A as a biomarker of brain microvascular damage in AD could have important implications for the treatment and prevention of this devastating disease, as microvascular retinal changes could be used as non-invasive outcome measures of the response to novel therapies against brain microvascular pathology.

## Methods

### Study subjects

The present study derives from the *Neuro-Ophthalmology Research At Fundació ACE* (NORFACE) cohort, which was founded in 2014 to investigate retinal biomarkers of AD and examine the relationship between retinal changes and different types of neurodegenerative disorders^20^. Consecutive patients evaluated due to cognitive decline at Ace Alzheimer Center Barcelona between January 2018 and March 2019 were enrolled in the present study. Participants were recruited from four different sources: (1) the Memory Clinic, an outpatient diagnostic unit for individuals with cognitive decline that has an agreement with the Catalan Public Health System, (2) Fundació ACE’s Open House Initiative^45^, a social program that assesses for free the cognitive status of individuals from the community without the need of a physician’s referral, (3) Fundació ACE Healthy Brain Initiative (FACEHBI)^46^, a research study with the goal of identifying biomarkers of preclinical AD in individuals with SCD and (4) the BIOFACE project^47,48^, a research study of novel biomarkers in early onset MCI. Inclusion criteria were: age ≥ 50-year old, presence of a consensus-based clinical diagnosis about the participants’ cognitive status, ability to complete the full ophthalmological exam and OCT scan (excluding those patients with severe dementia stages, equivalent to a Global Deteriorating Scale [GDS]^49^ score > 6).

### Clinical diagnostic groups

Study participants completed neurological, neuropsychological and social evaluations at Ace Alzheimer Center Barcelona. For each individual, a consensus-based diagnosis about the cognitive status was reached at the time of the study recruitment by a multidisciplinary team of professionals that included neurologists, neuropsychologists and social workers^50^. Cognitive assessment consisted of the Spanish version of the Mini-Mental State Examination (MMSE)^51,52^, the memory part of the Spanish version of the 7 Minutes test^53^, the Spanish version of the Neuropsychiatric Inventory Questionnaire (NPI-Q)^54^, the GDS^49^, the Clinical Dementia Rating Score (CDR)^55^, the Blessed Dementia Scale^56^ and a comprehensive neuropsychological battery of Fundació ACE (NBACE)^57,58^. Demographical information collected included age, gender and years of formal education. Past medical history collected included smoking habit, hypertension, diabetes mellitus, dyslipidemia, heart disease, stroke and chronic obstructive pulmonary disease (COPD). ADD was defined according to the NIA-AA criteria^59^. VaD was defined according to the NINDS-AIREN International Workshop Criteria^60^. MCI was defined using Petersen’s^61^ and the Cardiovascular health and cognition study criteria^62^. In particular, the MCI-AD group was characterized by memory impairment and absence of other comorbidities that could explain the cognitive decline (probable amnestic MCI^29^) with suspected underlying Alzheimer´s disease, while the MCI-Va group was defined as having a suspected underlying cerebrovascular etiology. The CU group included healthy controls and individuals with SCD. SCD refers to the perception of memory or other cognitive problems without impairment on standardized cognitive tests^63^. All individuals in the CU group had a CDR^55^ of 0, a preserved performance (score ≥ 27) on the MMSE^51,52^ and a strictly normal performance in the NBACE^57,58^.

### Neuro-ophthalmological evaluation

Study participants underwent a complete neuro-ophthalmological evaluation in parallel to the neurological assessment. The visit was performed by an optometrist and lasted about 20 min. Subjects had to be able to cooperate, obey simple instructions and sit still for a few minutes. The evaluation comprised: (1) review of past ophthalmological diseases, treatments and surgeries, (2) monocular visual acuity assessment with the participants wearing their habitual correction for refractive error using a pinhole occluder and the Early Treatment of Diabetic Retinopathy Study (ETDRS) chart^64,65^, (3) intraocular pressure (IOP) measurement by Icare tonometry^66^, and (4) swept source (SS) OCT scan. The visual acuity assessment was done the same way for all participants, regardless of their cognitive status and level of cooperation. Reduced visual acuity was defined as a standard LogMAR fraction scale ≤ 20/50 at 20 ft (equivalent to a fraction scale of 6/15 at 6 m and a decimal scale of 0.4) according to the Snellen scale^67^, which is in line with the North American guidelines of visual loss^68^. High IOP was defined as ≥ 24 mmHg using Icare Tonometry^66^. All assessments were carried out by a single optometrist and reviewed by a single ophthalmologist. Before beginning the study, the ophthalmologist trained the optometrist in the evaluation of OCT images, with the goal to differentiate normal images from abnormal findings. The ophthalmologist reviewed the ophthalmological history, ocular exam findings and OCT images from those cases in which OCT images were considered abnormal by the optometrist and came up with a diagnosis if appropriate. The ophthalmologist and neurologists were blind to each other’s diagnosis. Only OCT data from the right eye were analyzed. OCT-related exclusion criteria were the following: lack of collaboration in the neuro-ophthalmological exam or OCT scan, OCT data obtained only from the left eye, presence of OCT artefacts and diseases that could affect retinal measurements (e.g. open-angle glaucoma and other neuropathies maculopathies, prior retinal surgery, intraocular pressure [IOP] ≥ 24 mmHg, high myopia [< − 6Dp] or hyperopia [> + 6Dp] and optic nerve congenital abnormalities).

### Optical coherence tomography – angiography

**Figure 4:**
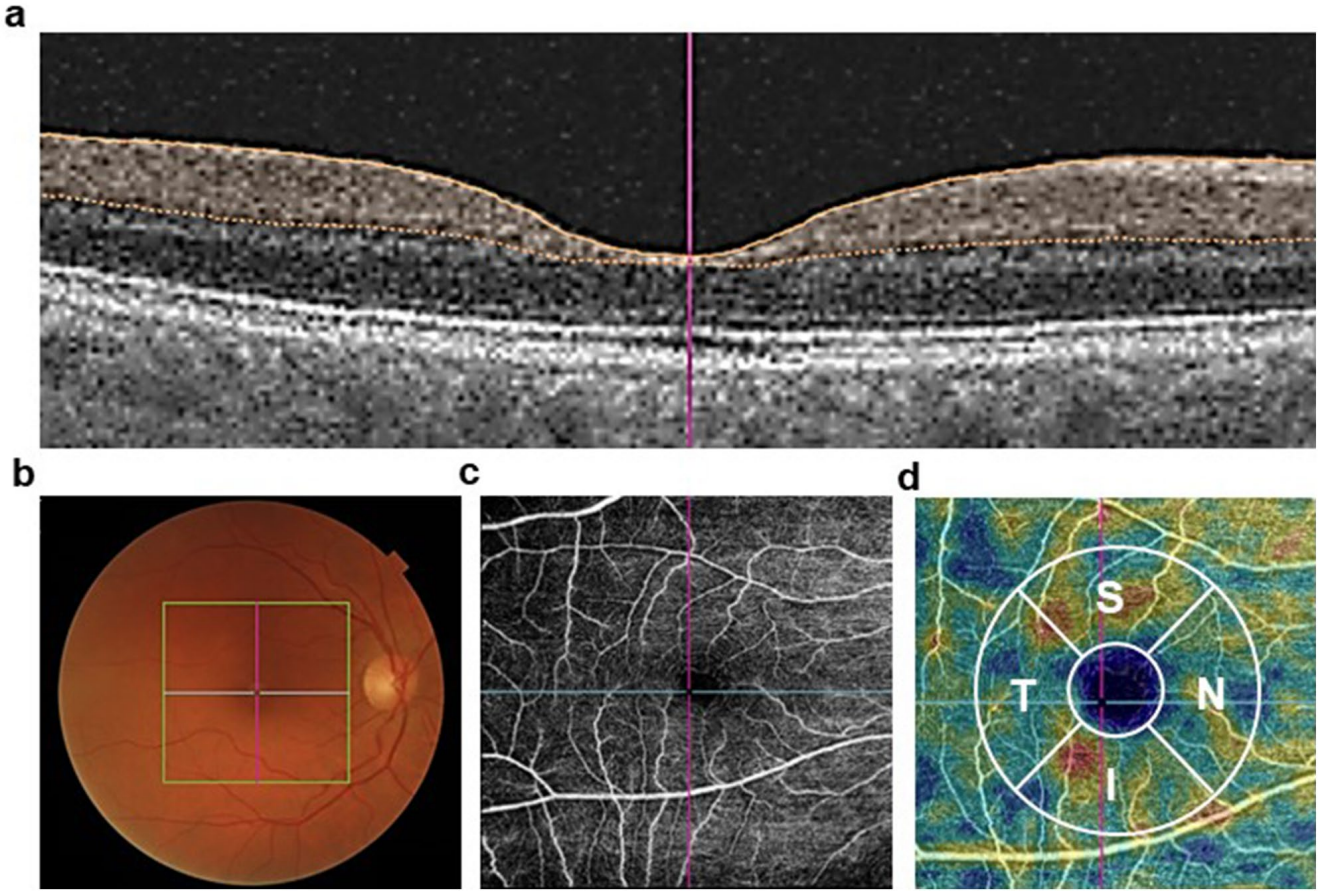
OCT-A imaging protocol. Limits of the superficial vascular plexus at the macular region (**a**). Grid on the macular region of the right eye (**b**). Vessels from the superficial vascular plexus at the macular region (**c**). The four macular quadrants analyzed (N, S, T and I) are depicted (**d**). C = center, N = nasal, S = superior, T = temporal and I = inferior.

Participants were imaged with a DRI OCT Triton—Swept Source (SS) OCT (Topcon Co. Tokyo, Japan). The OCT exam was completed in about 5–10 min. Both eyes were scanned separately. No pupil dilation was required. Data were analyzed with the OCT Angiography Ratio Analysis (OCTARA) processing software. An automatic segmentation method was employed to obtain the limits of the superficial vascular plexus (Fig. 4a), and the quantification of VD, expressed as the % of the macular area occupied by blood vessels. VD measures were obtained in a 6 × 6 mm area centered in the fovea (Fig. 4b, c). The central area (1 mm circle) was excluded from the analysis. The parafoveal area, defined by two concentric rings measuring 1 and 3 mm diameter, respectively, was subdivided into four quadrants: nasal, superior temporal and inferior (Fig. 4d). Only VD measures from the right eye were used for the analysis, as in previous manuscripts from our group^20–22^.

### Statistical analysis

Data processing and analysis were conducted using STATA v.15. All data were examined for normality, skew and restriction of range. All quantitative variables were normally distributed. Frequency analysis and measures of central tendency and dispersion were used to describe the demographic (age, sex, education) and clinical (hypertension, diabetes mellitus, dyslipidemia, heart disease, chronic obstructive pulmonary disease, stroke and smoking) variables among the five diagnostic groups. To summarize the distribution of these demographic and clinical variables among the five diagnostic groups, bivariate Analysis of Variance (ANOVA) and Pearson’s Chi2 tests were executed.

To identify demographic and clinical variables to be included as adjusting factors in a final multivariate model, two multinomial regression analysis were executed to determine their differential distribution among the five diagnostic groups. First, an analysis was performed for the demographics variables (age, sex and education), and then a second analysis was performed for the clinical variables (hypertension, diabetes mellitus, dyslipidemia, heart disease, chronic obstructive pulmonary disease, stroke, and smoking). In both analyses, the CU diagnostic group was considered the reference category.

The main analyses consisted of four multivariate regression analyses, one for every macular VD measure (nasal, superior, temporal and inferior quadrants), including the five diagnostic groups (CU, MCI-AD, MCI-Va, ADD and VaD) as discriminant factors and adjusting their effect by those factors that showed any significant effect in the two former multinomial regression analysis. The CU group was considered the reference category. Regression coefficients (the mean change in the outcome variable for one unit of change in the predictor variable while holding other predictors in the model constant), betas (the degree of change in the outcome variable for every 1-unit of change in the predictor variable) and t (assessing whether the beta coefficient is significantly different from zero) were reported.

As cardiovascular conditions are more strongly associated to cerebrovascular-related cognitive impairment (MCI-Va and VaD participants) than to the other diagnostic groups, regression models were rerun without those as adjusting factors, in order to avoid a potential collider bias effect.

We additionally performed sensitivity analysis to check the the effect of outlier cases (defined as ± 3 standard deviations of the VD mean for each macular quadrant) in the four multivariate regression analysis, using the CU group as the reference category.

To investigate whether a differential effect could be detected when considering sex, the previous four multivariate regression analyses were executed again, including now the interaction of diagnostic group x sex as the main factor of interest and the same covariates.

The association between MMSE scores and each of the four macular VD measurements was explored using a partial correlation, including the same covariates, for the whole sample and for each of the diagnostic groups separately.

Alpha level was set at 0.05.

### Ethical considerations

This study and its informed consent were approved by the ethics committee of the Hospital Clínic i Provincial de Barcelona in accordance with Spanish biomedical laws (Law 14/2007, July 3rd, about biomedical research; Royal Decree 1716/2011, November 18th) and followed the recommendations of the Declaration of Helsinki. All participants signed an informed consent (for those with moderate dementia stages, the informed consent was signed by their legal representative or family member).

## Supplementary Information

Supplementary Information.
