# Supplementary material for: Differences in macular vessel density in the superficial plexus across cognitive impairment: the NORFACE cohort
Source: Sci Rep. 2022 Oct 8;12:16938. doi: 10.1038/s41598-022-21558-w (PMC9547861; doi:10.1038/s41598-022-21558-w)
Supplement: Supplementary file 1 — Supplementary Information. [file 41598_2022_21558_MOESM1_ESM.pdf]

## Supplementary Information

**Supplementary Table S1. Multinomial regression analysis of the distribution of age, sex and education among diagnostic groups**

| Diagnostic groups comparisons | Dependent variables | Relative risk | Standard error | z | Significance | 95% confidence interval |
|---|---|---|---|---|---|---|
| CU vs MCI-AD | Age | 1.15 | 0.02 | 6.90 | <0.01* | 1.10 - 1.20 |
| | Sex | 0.28 | 0.09 | -3.92 | <0.01* | 0.15 - 0.53 |
| | Education | 0.84 | 0.03 | -4.52 | <0.01* | 0.78 - 0.91 |
| CU vs MCI-Va | Age | 1.17 | 0.02 | 7.34 | <0.01* | 1.12 - 1.22 |
| | Sex | 0.34 | 0.11 | -3.30 | <0.01* | 0.18 - 0.64 |
| | Education | 0.82 | 0.03 | -4.93 | <0.01* | 0.76 - 0.89 |
| CU vs ADD | Age | 1.24 | 0.03 | 10.41 | <0.01* | 1.19 - 1.29 |
| | Sex | 0.67 | 0.21 | -1.26 | <0.01* | 0.36 - 1.25 |
| | Education | 0.80 | 0.03 | -5.88 | <0.01* | 0.74 - 0.86 |
| CU vs VaD | Age | 1.24 | 0.03 | 7.96 | <0.01* | 1.18 - 1.31 |
| | Sex | 0.45 | 0.18 | -1.97 | <0.01* | 0.20 - 1.00 |
| | Education | 0.79 | 0.04 | -4.79 | <0.01* | 0.71 - 0.87 |

Abbreviations: ADD = probable Alzheimer´s disease dementia; CU = cognitively unimpaired; MCI-AD = mild cognitive impairment due to Alzheimer´s disease; MCI-Va = mild cognitive impairment due to cerebrovascular pathology; VaD = vascular dementia.

Significance was set up at p<0.05.

**Supplementary Table S2. Multinomial regression analysis of the distribution of cardiovascular medical conditions among diagnostic groups**

| Diagnostic group comparisons | Dependent variables | Relative risk | Standard error | z | Significance | 95% confidence interval |
|---|---|---|---|---|---|---|
| CU vs MCI-AD | Hypertension | 1.67 | 0.47 | 1.83 | 0.07 | 0.96 - 2.89 |
| | Diabetes mellitus | 1.06 | 0.48 | 0.12 | 0.90 | 0.43 - 2.57 |
| | Dyslipidemia | 1.33 | 0.36 | 1.05 | 0.30 | 0.78 - 2.28 |
| | Heart disease | 0.26 | 0.80 | 2.28 | 0.02* | 1.12 - 4.54 |
| | COPD | 1.10 | 0.51 | 0.19 | 0.85 | 0.44 - 2.74 |
| | Stroke | 1.16 | 0.78 | 0.22 | 0.83 | 0.31 - 4.32 |
| | Smoking | 0.31 | 0.21 | -1.75 | 0.08 | 0.39 - 0.87 |
| CU vs MCI-Va | Hypertension | 2.67 | 0.80 | 3.27 | <0.01* | 1.48 - 4.81 |
| | Diabetes mellitus | 2.93 | 1.24 | 2.53 | 0.01* | 1.27 - 6.74 |
| | Dyslipidemia | 0.75 | 0.22 | -0.98 | 0.33 | 0.42 - 1.33 |
| | Heart disease | 1.89 | 0.69 | 1.75 | 0.08 | 0.93 - 3.87 |
| | COPD | 1.90 | 0.84 | 1.44 | 0.15 | 0.79 - 4.54 |
| | Stroke | 5.72 | 3.31 | 3.01 | 0.01* | 1.84 - 17.81 |
| | Smoking | 1.15 | 0.57 | 0.28 | 0.78 | 0.44 - 3.03 |
| CU vs ADD | Hypertension | 2.71 | 0.65 | 4.13 | <0.01* | 1.69 - 4.34 |
| | Diabetes mellitus | 1.12 | 0.45 | 0.29 | 0.77 | 0.51 - 2.45 |
| | Dyslipidemia | 1.00 | 0.24 | -0.01 | 0.99 | 0.63 - 1.59 |
| | Heart disease | 2.03 | 0.65 | 2.20 | 0.03* | 1.08 - 3.81 |
| | COPD | 0.94 | 0.39 | -0.15 | 0.88 | 0.42 - 2.11 |
| | Stroke | 1.37 | 0.81 | 0.54 | 0.59 | 0.43 - 4.36 |
| | Smoking | 0.63 | 0.28 | -1.04 | 0.30 | 0.26 - 1.51 |
| CU vs VaD | Hypertension | 5.40 | 2.38 | 3.83 | <0.01* | 2.28 - 12.80 |
| | Diabetes mellitus | 4.69 | 2.20 | 3.30 | <0.01* | 1.87 - 11.76 |
| | Dyslipidemia | 0.68 | 0.25 | -1.04 | 0.30 | 0.33 - 1.41 |
| | Heart disease | 3.00 | 1.23 | 2.67 | <0.01* | 1.34 - 6.71 |
| | COPD | 1.77 | 0.96 | 1.05 | 0.29 | 0.61 - 5.13 |
| | Stroke | 6.68 | 4.18 | 3.03 | <0.01* | 1.96 - 22.76 |
| | Smoking | 1.08 | 0.71 | 0.12 | 0.91 | 0.30 - 3.91 |

Abbreviations: ADD = probable Alzheimer´s disease dementia; COPD = chronic obstructive pulmonary disease; CU = cognitively unimpaired; MCI-AD = mild cognitive impairment due to Alzheimer´s disease; MCI-Va = mild cognitive impairment due to cerebrovascular pathology; VaD = vascular dementia.

Significance was set up at $p<0.05$.

**Supplementary Table S3. Multivariate regression analysis of macular VD measurements without cardiovascular conditions as adjusting variables.**

| **Diagnostic groups comparisons** | **Dependent variables** | **Coefficient** | **t** | **Significance** | **Beta** |
|---|---|---|---|---|---|
| CU vs MCI-AD | VD Nasal | 0.89 | 1.01 | 0.31 | 0.06 |
| | VD Superior | 0.60 | -0.30 | 0.76 | -0.03 |
| | VD Temporal | 1.75 | 2.34 | 0.02* | 0.13 |
| | VD Inferior | -1.55 | -1.48 | 0.14 | -0.08 |
| CU vs MCI-Va | VD Nasal | 0.02 | 0.02 | 0.99 | <0.01 |
| | VD Superior | -0.31 | -0.30 | 0.76 | -0.02 |
| | VD Temporal | 0.49 | 0.63 | 0.53 | 0.03 |
| | VD Inferior | -2.15 | -1.98 | 0.04* | -0.11 |
| CU vs ADD | VD Nasal | 0.28 | 0.33 | 0.74 | 0.02 |
| | VD Superior | 0.52 | 0.55 | 0.59 | 0.04 |
| | VD Temporal | 1.21 | 1.66 | 0.10 | 0.11 |
| | VD Inferior | -0.87 | -0.85 | 0.40 | -0.06 |
| CU vs VaD | VD Nasal | 0.64 | 0.56 | 0.57 | 0.03 |
| | VD Superior | 1.41 | 1.12 | 0.27 | 0.06 |
| | VD Temporal | 1.37 | 1.43 | 0.15 | 0.07 |
| | VD Inferior | 0.63 | 0.47 | 0.64 | 0.02 |

The multivariate regression analysis included the following adjusting factors: age, sex and years of education.

Abbreviations: ADD = probable Alzheimer´s disease dementia; CU = cognitively unimpaired; MCI-AD = mild cognitive impairment due to Alzheimer´s disease; MCI-Va = mild cognitive impairment due to cerebrovascular pathology; VaD = vascular dementia; VD = vessel density.

Significance was set up at $p<0.05$.

**Supplementary Table S4. Multivariate regression analysis of the interaction of sex and diagnosis in predicting macular VD measurements**

| Diagnostic group comparisons | Dependent variables | Coefficient | t | Significance | Beta |
|---|---|---|---|---|---|
| CU vs MCI-AD | VD Nasal | 0.82 | 0.50 | 0.62 | 0.04 |
| | VD Superior | -1.74 | -0.95 | 0.35 | -0.07 |
| | VD Temporal | 1.36 | 0.98 | 0.33 | 0.07 |
| | VD Inferior | -1.68 | -0.87 | 0.39 | -0.06 |
| CU vs MCI-Va | VD Nasal | -1.41 | -0.84 | 0.40 | -0.06 |
| | VD Superior | 0.72 | 0.39 | 0.70 | 0.03 |
| | VD Temporal | -0.97 | -0.68 | 0.49 | -0.05 |
| | VD Inferior | -0.89 | -0.45 | 0.65 | -0.03 |
| CU vs ADD | VD Nasal | 0.11 | 0.07 | 0.94 | 0.01 |
| | VD Superior | -0.81 | -0.49 | 0.62 | -0.05 |
| | VD Temporal | 0.12 | 1.83 | 0.07 | 0.14 |
| | VD Inferior | -0.02 | -0.01 | 0.99 | -0.01 |
| CU vs VaD | VD Nasal | 2.34 | 2.09 | 0.26 | 0.08 |
| | VD Superior | 0.34 | 0.15 | 0.88 | 0.01 |
| | VD Temporal | 3.23 | 1.83 | 0.07 | 0.14 |
| | VD Inferior | -0.79 | -0.32 | 0.75 | -0.02 |

The multivariate regression analysis included the following adjusting factors: age, years of education, hypertension, diabetes mellitus, heart disease and stroke.

Abbreviations: ADD = probable Alzheimer´s disease dementia; CU = cognitively unimpaired; MCI-AD = mild cognitive impairment due to Alzheimer´s disease; MCI-Va = mild cognitive impairment due to cerebrovascular pathology; VaD = vascular dementia; VD = vessel density.

Significance was set up at $p<0.05$.

**Supplementary Table S5. Multivariate regression analysis of macular VD measurements with pairwise comparisons using Tukey correction**

| Diagnostic group comparisons | Dependent variables | Mean difference | Standard error | t | Significance | 95% confidence interval |
|---|---|---|---|---|---|---|
| CU vs MCI-AD | VD Nasal | 0.87 | 0.89 | 0.98 | 0.86 | -1.55 -3.29 |
| | VD Superior | 0.64 | 0.99 | 0.65 | 0.97 | -2.07- 3.36 |
| | VD Temporal | 1.77 | 0.75 | 2.36 | 0.13 | -0.28 - 3.83 |
| | VD Inferior | -1.59 | 1.04 | -1.52 | 0.55 | -4.45 -1.27 |
| CU vs MCI-Va | VD Nasal | 0.07 | 0.93 | 0.07 | 1.00 | -2.48 - 2.62 |
| | VD Superior | -0.22 | 1.04 | -0.21 | 1.00 | -3.07 - 2.64 |
| | VD Temporal | 0.50 | 0.79 | 0.64 | 0.97 | -1.66 - 2.67 |
| | VD Inferior | -2.58 | 1.10 | -2.34 | 0.13 | -5.58 - 0.43 |
| CU vs ADD | VD Nasal | 0.27 | 0.86 | 0.31 | 1.00 | -2.09 - 2.62 |
| | VD Superior | 0.57 | 0.96 | 0.59 | 0.98 | 2.07 - 3.21 |
| | VD Temporal | 1.23 | 0.73 | 1.68 | 0.45 | -0.77 - 3.23 |
| | VD Inferior | -1.04 | 1.02 | -1.03 | 0.84 | -3.82 - 1.74 |
| CU vs VaD | VD Nasal | 0.85 | 1.17 | 0.73 | 0.95 | -2.34 - 4.04 |
| | VD Superior | 1.40 | 1.31 | 1.07 | 0.82 | -2.18 - 4.97 |
| | VD Temporal | 1.29 | 0.99 | 1.30 | 0.69 | -1.42 - 4.00 |
| | VD Inferior | 0.09 | 1.38 | 0.07 | 1.00 | -3.67 - 3.86 |
| MCI-AD vs MCI-Va | VD Nasal | -0.80 | 0.83 | -0.97 | 0.87 | -3.07 - 1.46 |
| | VD Superior | -0.86 | 0.93 | -0.93 | 0.89 | -3.40 - 1.68 |
| | VD Temporal | -1.27 | 0.70 | -1.81 | 0.37 | -3.19 - 0.65 |
| | VD Inferior | -0.99 | 0.98 | -1.01 | 0.85 | -3.66 - 1.68 |
| MCI-AD vs ADD | VD Nasal | -0.60 | 0.71 | -0.85 | 0.91 | -2.54 - 1.33 |
| | VD Superior | -0.07 | 0.79 | -0.09 | 1.00 | -2.24 - 2.09 |
| | VD Temporal | -0.55 | 0.60 | -0.91 | 0.89 | -2.19 - 1.09 |
| | VD Inferior | 0.55 | 0.83 | 0.66 | 0.97 | -1.73 - 2.83 |
| MCI-AD vs VaD | VD Nasal | -0.02 | 1.05 | -0.02 | 1.00 | -2.89 - 2.85 |
| | VD Superior | 0.75 | 1.17 | 0.64 | 0.97 | -2.46 - 3.96 |
| | VD Temporal | -0.48 | 0.89 | -0.54 | 0.98 | -2.92 - 1.95 |
| | VD Inferior | 1.68 | 1.24 | 1.36 | 0.65 | -1.70 - 5.07 |
| MCI-Va vs ADD | VD Nasal | 0.20 | 0.73 | 0.27 | 1.00 | -1.79 - 2.18 |
| | VD Superior | 0.79 | 0.81 | 0.97 | 0.87 | -1.44 - 3.01 |
| | VD Temporal | 0.72 | 0.62 | 1.17 | 0.77 | -0.96 - 2.41 |
| | VD Inferior | 1.54 | 0.86 | 1.80 | 0.38 | -0.80 - 3.88 |
| MCI-Va vs VaD | VD Nasal | 0.78 | 1.02 | 0.76 | 0.94 | -2.02 - 3.58 |
| | VD Superior | 1.61 | 1.15 | 1.40 | 0.63 | -1.53 - 4.75 |
| | VD Temporal | 0.79 | 0.87 | 0.90 | 0.90 | -1.59 - 3.16 |
| | VD Inferior | 2.67 | 1.21 | 2.21 | 0.18 | -0.63 - 5.98 |
| ADD vs VaD | VD Nasal | 0.58 | 0.94 | 0.62 | 0.97 | -2.00 - 3.16 |
| | VD Superior | 0.82 | 1.06 | 0.78 | 0.94 | -2.06 - 3.71 |
| | VD Temporal | 0.06 | 0.80 | 0.08 | 1.00 | -2.12 - 2.25 |
| | VD Inferior | 1.13 | 1.11 | 1.02 | 0.85 | -1.90 - 4.17 |

Pairwise comparisons among diagnostic groups using Tukey correction were performed.

Abbreviations: ADD = probable Alzheimer´s disease dementia; CU = cognitively unimpaired; MCI-AD = mild cognitive impairment due to Alzheimer´s disease; MCI-Va = mild cognitive impairment due to cerebrovascular pathology; VaD = vascular dementia; VD = vessel density.

Significance was set up at $p<0.05$.
